# A novel Mutein of TNFα Containing the Arg-Gly-Asp Sequence Shows Reduced Toxicity in Intestine

**DOI:** 10.1155/S096293519400013X

**Published:** 1994

**Authors:** H. Shikama, K. Miyata, N. Sakae, K. Kuroda, K. Nishimura, S. Yotsuya, M. Kato

**Affiliations:** 1 Biochemistry Research Laboratory Central Research Institute Ishihara Sangyo Kaisha Ltd 2-3-1, Nishi-shibukawa, Kusatsu, Shiga 525 Japan; 2 Environmental Sciences Laboratory Central Research Institute Ishihara Sangyo Kaisha Ltd 2-3-1, Nishi-shibukawa, Kusatsu, Shiga 525 Japan

## Abstract

The effects of human tumour necrosis factor-α (TNFα), or its mutein (F4168) having the cell adhesive Arg-Gly-Asp sequence at the N-terminus, on intestinal injury, were examined. Histopathological examination revealed that an intravenous injection of TNFα resulted in marked haemorrhage or oedema in the caecum of rats, whereas F4168 showed no such effects even at the same therapeutic dose. Moreover, the number of neutrophils that adhered to endothelial cells or infiltrated the mucosal tissue was much higher after TNFα injection compared with F4168 *in vivo*. The enhanced adhesion of neutrophils on to human umbilical vein endothelial cells also occurred when the latter were pre-stimulated with TNFα but not with F4168 *in vitro*. The expression of the cell adhesion molecules including endothelial leukocyte adhesion molecule-1 or intercellular adhesion molecule-1 on F4168- stimulated human umbilical vein endothelial ceils was significantly lower than that stimulated with TNFα. These results suggest that the Arg-Gly-Asp sequence introduced into the TNFα molecule abrogates the side effect of this cytokine such as tissue injury or shock, and that F4168 could be useful for systemic therapy.


Mediators of Inflammation 3, 111–116 (1994)

### A novel mutein of TNFα containing the Arg–Gly–Asp sequence shows reduced toxicity in intestine

H. Shikama,[1,CA] K. Miyata,[1] N. Sakae,[1] K. Kuroda,[1] K. Nishimura,[1] S. Yotsuya[2] and M. Kato[1]

[1]Biochemistry Research Laboratory, and [2]Environmental Sciences Laboratory, Central Research Institute, Ishihara Sangyo Kaisha Ltd, 2-3-1, Nishi-shibukawa, Kusatsu, Shiga 525, Japan

[CA] Corresponding Author

**The effects of human tumour necrosis factor-α (TNFα), or its mutein (F4168) having the cell adhesive Arg–Gly–Asp sequence at the N-terminus, on intestinal injury, were examined. Histopathological examination revealed that an intravenous injection of TNFα resulted in marked haemorrhage or oedema in the caecum of rats, whereas F4168 showed no such effects even at the same therapeutic dose. Moreover, the number of neutrophils that adhered to endothelial cells or infiltrated the mucosal tissue was much higher after TNFα injection compared with F4168 *in vivo*. The enhanced adhesion of neutrophils on to human umbilical vein endothelial cells also occurred when the latter were pre-stimulated with TNFα but not with F4168 *in vitro*. The expression of the cell adhesion molecules including endothelial leukocyte adhesion molecule-1 or intercellular adhesion molecule-1 on F4168-stimulated human umbilical vein endothelial cells was significantly lower than that stimulated with TNFα. These results suggest that the Arg–Gly–Asp sequence introduced into the TNFα molecule abrogates the side effect of this cytokine such as tissue injury or shock, and that F4168 could be useful for systemic therapy.**

**Key words:** Cell adhesion molecule, Endothelial cell, Neutrophil, Tissue injury, Tumour necrosis factor

### Introduction

TNFα is a multi-functional cytokine which was originally identified as a serum factor that induced necrotic responses of solid tumours *in vivo*.[1] Although TNFα exerts antitumour effects against various tumour cell lines,[1–3] it also has hypotensive[4,5] or tumour metastasis-enhancing effects.[6–8] Moreover, TNFα induces shock and injury including intestinal necrosis in rats or mice when it is administered with or without LPS.[4,5,9–11] Thus, for clinical applications, it is necessary to reduce these side-effects of TNFα while maintaining its antitumour potential. It was demonstrated previously that a TNF mutein (F4236) having the laminin derived cell-adhesive peptide (YIGSR) did not enhance experimental pulmonary metastasis in contrast to wild-type TNFα.[6] An Arg–Gly–Asp (RGD) containing mutein (F4168) also showed similar properties. Here, the effect of F4168 on inflammatory reactions, including shock and tissue injury in rats, has been examined.

### Materials and Methods

*Construction of expression plasmids:* The human TNFα gene prepared with synthetic oligonucleotides was used to construct the expression plasmid pKF4102 for wild-type TNFα. The expression plasmid pKF4168 was used for the mutein (F4168) having the tripeptide Arg–Gly–Asp (RGD) near the N-terminus of human TNFα. This was introduced into the wild-type TNFα gene by site-directed mutagenesis.[12] The construction of pKF4168 involved the substitution of Gly–Asp for the $^{5}$Thr-$^{6}$Pro of wild-type TNFα. Both pKF4102 and pKF4168 have the *tac* promoter.

*Production and purification of TNFα and F4168:* The expression plasmids were induced by adding IPTG (isopropyl-β-D-thio-galactopyranoside) into the *Escherichia coli* (K12 JM103) culture medium (M9). These proteins were expressed in *E. coli*, extracted by sonication and purified to homogeneity by sequential anion exchange chromatography using Sepabeads FP-DA13 (Mitsubishi Chemical Industries Co., Tokyo, Japan) and Mono Q (Pharmacia LKB Biotechnology, Uppsala, Sweden). The specific activity of both TNFα and F4168 was $3 \times 10^7$ units/mg protein. The protein content was determined by a dye-binding method.[13]

*The effect of TNFα or F4168 on tissue injury:* Male Sprague–Dawley rats (220 ± 25 g; Crj; CD) were housed in standard cages and given food and water *ad libitum* in a temperature controlled room (24 ± 1°C) with a 12 h dark/light cycle until surgery. TNFα and F4168 dissolved in saline were intravenously injected at a dose of 20 μg/500 μl/rat. The animals were sacrificed 6 h after injection using $CO_2$

© 1994 Rapid Communications of Oxford Ltd gas. A complete post-mortem examination including sampling of digestive organs for histopathological examination was performed. The digestive organs were fixed in 10% neutral buffered formalin, processed by conventional methods, embedded in paraffin, cut into 4 μm thick sections, then stained with haematoxylin and eosin.

*Isolation and culture of human umbilical vein endothelial cells:* Human umbilical vein endothelial cells (HUVEC) were prepared by collagenase digestion of umbilical veins essentially according to the method of Jaffe *et al.*[14] Isolated cells were routinely cultured in growth media consisting of MCDB107 (Kyokuto Pharmaceutical Industrial Co. Ltd, Japan) supplemented with endothelial cell growth supplement (50 μg/ml; Collaborative Research Inc.), heparin (50 μg/ml; Sigma), penicillin (100 units/ml), streptomycin (100 μg/ml) and 10% FBS (Gibco). Cultures were free of contaminating fibroblast and smooth muscle cell as assessed by the expression of Factor VIII-related antigen and uptake of acetylated low density lipoprotein and were used up to passage five. The cells were harvested from 35 mm gelatin precoated tissue culture plates (Corning) by exposure to 0.125% trypsin with 1 mM EDTA for 2–3 min. Cells ($1 \times 10^4$/well) were seeded onto 96-well plates (Corning) which were precoated with 0.5% gelatin for 30 min at 37°C. Cells were cultured for a further 3–4 days before the assay.

*Neutrophil adhesion assay:* Neutrophils were isolated from the peripheral blood of healthy adult volunteers by density gradient centrifugation with Polymorphprep™ (Nycomed Pharma AS, Oslo, Norway). Confluent HUVEC were incubated with 0.1 ng/ml TNFα or F4168 for 4 h at 37°C. The wells were then gently washed twice with MCDB 107 supplemented with 10% FBS. Neutrophils were added ($2 \times 10^5$ cells/100 μl/well) onto the factor stimulated HUVEC and allowed to attach for 30 min at 37°C. The non-adherent cells were then removed and the wells were washed three times with phosphate buffered saline (PBS). The adherent cells were fixed in 3.7% buffered formalin. The number of neutrophils that adhered to the HUVEC was counted in five fields (1 $mm^2$) for each well.

*Antibodies and other reagents:* Mouse IgG anti-human ELAM-1 (BBA1) and mouse IgG anti-human ICAM-1 (BBA3) were obtained from British Bio-technology Products Ltd. Anti-mouse IgG(H+L) and the avidin-biotinylated peroxidase kit were from Vector Laboratories, Inc.

*ELISA:* ELAM-1 and ICAM-1 expression on HUVEC was measured using an ELISA. Confluent cultures of HUVEC were stimulated with several concentrations of TNFα or mutein in culture medium. HUVEC were fixed with 3.7% buffered formalin, then washed with 0.5% BSA in PBS (BSA buffer) four times. Fc receptors were blocked by incubating with horse serum (diluted 1:50 in BSA buffer) for 1 h at 37°C. The cells were then washed and incubated with mouse anti-human ELAM-1 (diluted 1:2 000) or mouse anti-human ICAM-1 (diluted 1:1 000) with BSA buffer for 1 h at 37°C. The cells were washed and incubated with biotinylated horse anti-mouse IgG with BSA buffer for 1 h, followed by another wash and incubation with avidin-biotinylated peroxidase. Binding was assessed by adding 100 μl of 0.1 mg/ml tetramethylbenzidine with 0.3% $H_2O_2$ in distilled water. The reaction was stopped with 4 N sulphuric acid, and plates were read on an ELISA plate reader (Bio-Rad) at an OD of 450 nm. The degree of specific mAb binding was calculated by subtracting the non-specific binding of the negative control from all test values.

### Results

The acute toxicity caused by the intravenous injection of TNFα or F4168 in rats has been examined. Since the maximal hypotensive effect in rats and serious tissue injury in mice were observed 6 to 8 h after TNFα injection, the rats were killed for necropsy and histopathological examinations 6 h after TNFα or F4168 injection.

At necropsy, several gross abnormalities of the caecum including marked thickening of the walls or focal haemorrhage were apparent in rats injected with TNFα. Histopathological sections through the caecum 6 h after TNFα injection (20 μg) revealed an inflammatory response with haemorrhage in the lamina propria mucosae or infiltration of leukocytes (Fig. 1A). The venule of the tunica submucosa was dilated, and invasion of leukocytes was also observed. A detailed histopathological examination of the venule revealed the adhesion of neutrophils on the vessel wall or infiltration into the surrounding mucosal tissues (Fig. 1B). In contrast, rats injected with F4168 had a reduced inflammatory response compared with the TNFα administered group. Haemorrhage was rarely observed (Fig. 1D), and the number of lymphocytes or neutrophils adhering or infiltrating in mucosa was relatively small in the F4168 administered group (Fig. 1E). Table 1 shows the comparison of the effects of TNFα and F4168 on several histopathological findings. Similar results were obtained for the duodenum of mice injected with TNFα or F4168 (data not shown).

The effect of TNFα or F4168 on the neutrophil adhesion to HUVEC *in vitro* (Fig. 2) also was examined. Human neutrophils were overlaid on HUVEC prestimulated with 0.1 ng/ml of TNFα or F4168, and the adhesion during 30 min incubation at 37°C was

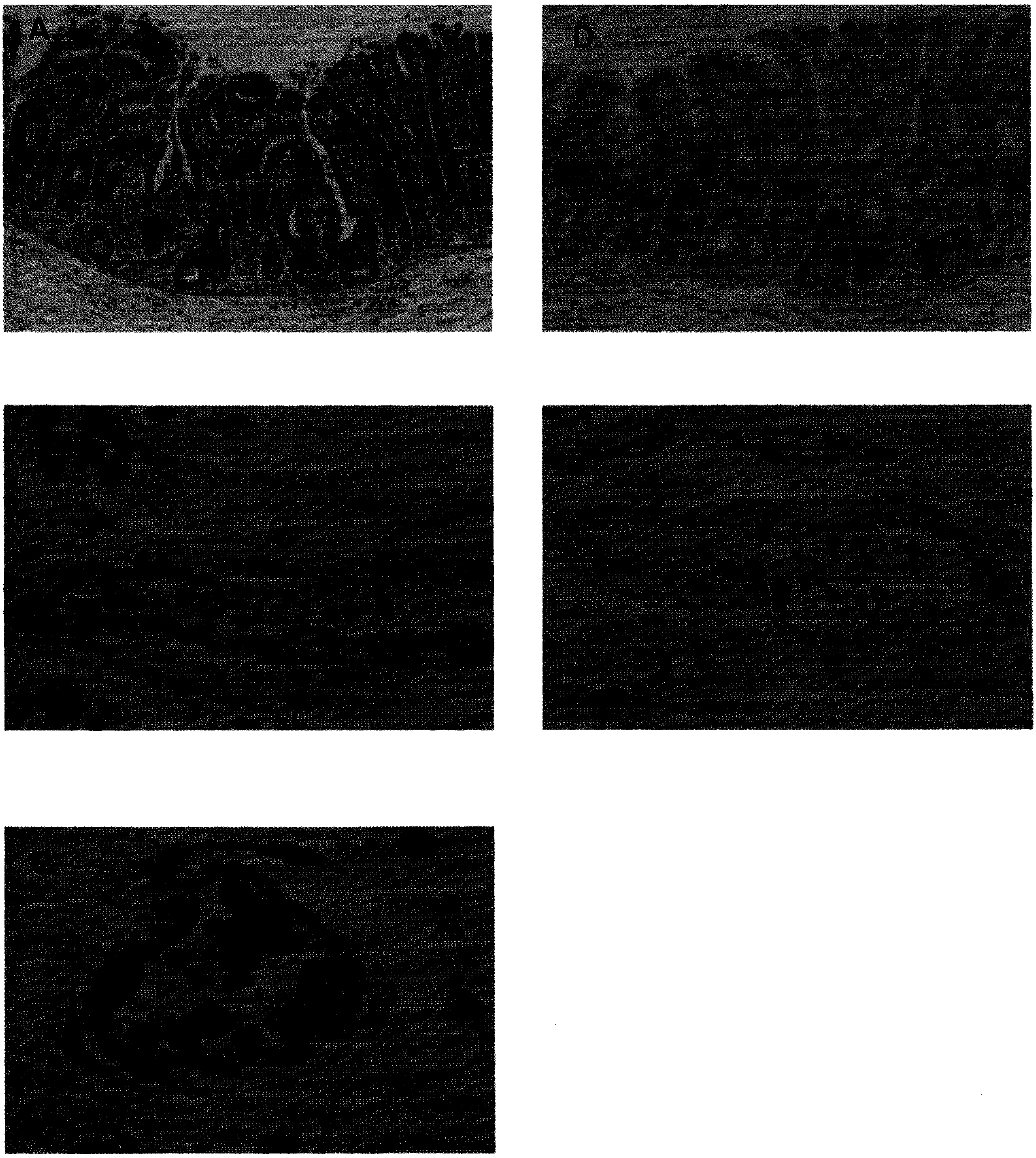

Fig. 1. The histopathological appearance of the caecum in Sprague–Dawley rats given TNFα or F4168. TNFα (20 μg/rat) (Panels A, B and C) or F4168 (20 μg/rat) (Panels D and E) was administered intravenously. Six hours later, the rats were sacrificed for histopathological examination. A and D, × 100; B and E, × 400; C, × 800. There is a large number of neutrophils adhering to the vascular endothelium.

evaluated. The number of adherent neutrophils onto the F4168-stimulated HUVEC was significantly lower than that in TNFα (154 ± 30 *vs.* 236 ± 49 cells/mm$^2$, $p < 0.01$). Similar results were also obtained when HUVEC were prestimulated with 1 ng/ml of each factor (data not shown).

The TNFα or F4168 induced ELAM-1 on HUVEC were next examined. Although the initial expression rate of ELAM-1 by TNFα or F4168 (0.1 ng/ml, each) was almost the same, the maximal expression (4 or 6 h after stimulation) by F4168 was significantly lower than that by TNFα (Fig. 3). Moreover, the ELAM-1 induction by TNFα was dose dependent at concentrations of 0.01 to 10 ng/ml (Fig. 4). F4168, however, induced a relatively low level of ELAM-1 expression compared with that by TNFα at any

**Table 1.** Distribution of histopathological findings in the caecum of rats

| Findings | Ileocaecum | | | Corpus | | |
|---|---|---|---|---|---|---|
| | Saline | TNFα | F4168 | Saline | TNFα | F4168 |
| Diffuse dilatation of blood capillaries in lamina propria mucosae | 0[a] | 0.9 | 0.3 | 0 | 0.2 | 0.3 |
| Focal erosion | 0 | 0.3 | 0 | 0.2 | 0.5 | 0.1 |
| Focal necrosis or disappearance of epithelial cells | 0 | 1.3 | 0.5 | 0 | 0.6 | 0.2 |
| Focal necrosis in lamina propria mucosae | 0 | 0.9 | 0.4 | 0 | 0.4 | 0.2 |
| Focal infiltration of neutrophil cells in lamina propria mucosae | 0.1 | 0.8 | 0.4 | 0 | 0.7 | 0.3 |
| Focal infiltration of neutrophil cells in tunica submucosa | 0.1 | 0.5 | 0.3 | 0 | 0.6 | 0.3 |
| Focal haemorrhage in lamina propria mucosae | 0 | 1.1 | 0.5 | 0 | 0.9 | 0.5 |
| Focal haemorrhage in tunica submucosa | 0 | 0.3 | 0.1 | 0 | 0.1 | 0 |
| Oedema in lamina propria mucosae | 0.1 | 0.7 | 0.4 | 0 | 0.3 | 0.2 |
| Oedema in tunica submucosa | 0 | 1.3 | 1.0 | 0.2 | 1.9 | 0.9 |

Sacrificed 6 h after injection ($n = 8$ for TNFα or F4168 injected group, $n = 6$ for saline control group). [a]Severity score—the sum of the individual intensity of histopathological scores (minimal, 0.5; slight, 1; moderate, 2; marked, 3) divided by the total number of rats per group.

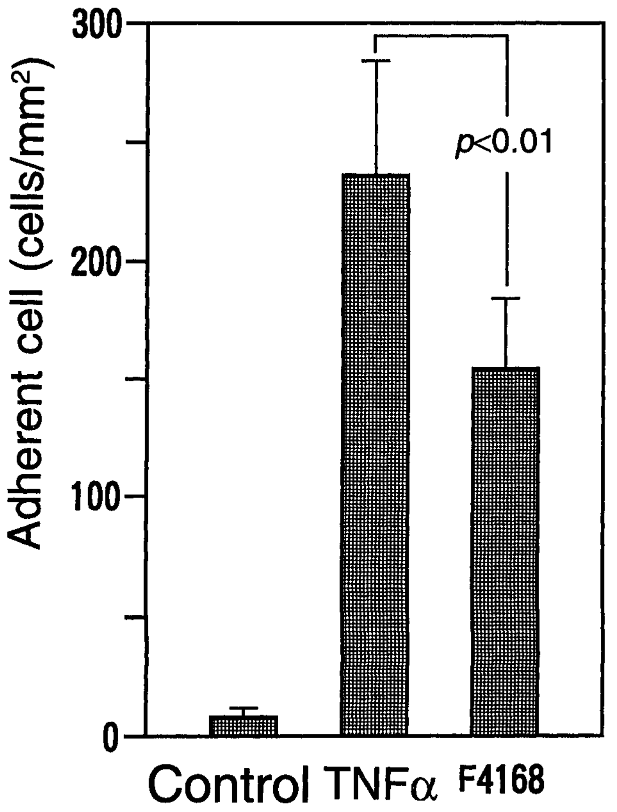

Fig. 2. Adhesion of isolated neutrophils to TNFα- or F4168-stimulated HUVEC. HUVEC were stimulated with TNFα or F4168 (0.1 ng/ml, each) for 4 h at 37°C. Neutrophils ($2 \times 10^5$ cells) were then plated and allowed to adhere for 30 min and counted. Bars indicate the mean ± S.D., and significance was determined by Student's *t*-test.

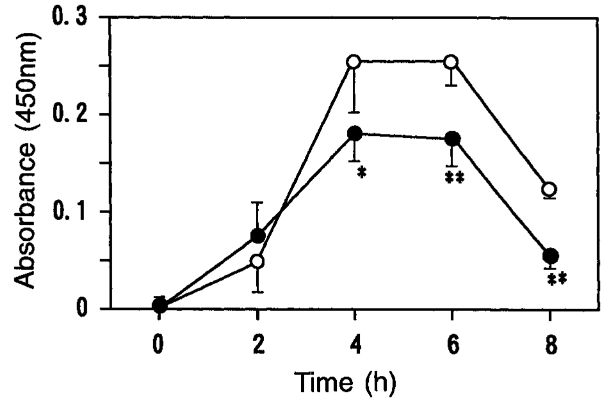

Fig. 3. Time course of ELAM-1 induction on HUVEC. HUVEC were stimulated with 0.1 ng/ml of TNFα, (unfilled circles) or F4168 (filled circles). At the indicated times, ELAM-1 induction was determined by ELISA. Bars indicate the mean ± S.D., and significance was determined by Student's *t*-test (*$p < 0.05$; **$p < 0.001$).

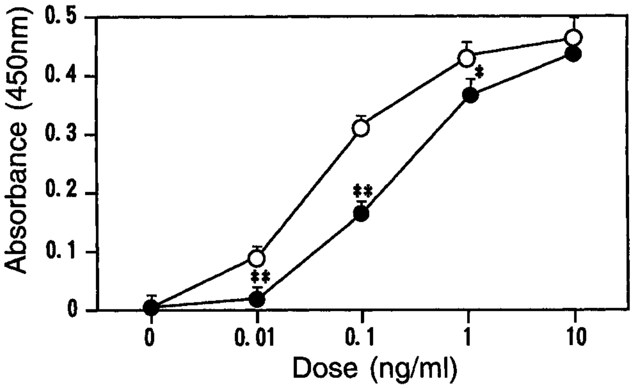

Fig. 4. Dose dependent up-regulation of ELAM-1 on HUVEC. The cell surface ELAM-1 level was increased by stimulating HUVEC with TNFα (unfilled circles) or F4168 (filled circles) at doses ranging from 0.001 to 10 ng/ml for 4 h. Bars indicate the mean ± S.D., and significance was determined by Student's *t*-test (*$p < 0.005$; **$p < 0.001$).

concentration tested except 10 ng/ml (Fig. 4). Similar results were also obtained with respect to ICAM-1 (Fig. 5B), suggesting that the RGD sequence in F4168 reduces the enhanced ELAM-1 or ICAM-1 inducibility of TNFα.

Although the GRGDS peptide did not affect the TNFα mediated enhancement of ELAM-1 expression (Fig. 5A), it reproducibly (but not significantly) reduced the TNFα mediated ICAM-1 expression at a dose of 3 or 100 pg/ml (6 and 200 pM, respectively) (Fig. 5B). On the other hand, F4168 induced significantly lower levels of ICAM-1 expression compared with TNFα at a dose of 0.1 ng/ml (6 pM). These data confirm the effectiveness of the RGD sequence in F4168 in reducing the enhanced induction of cell adhesion molecules.

### Discussion

It is postulated that the antitumour activity of TNFα is completed as the result of the 'primary' and 'sec-

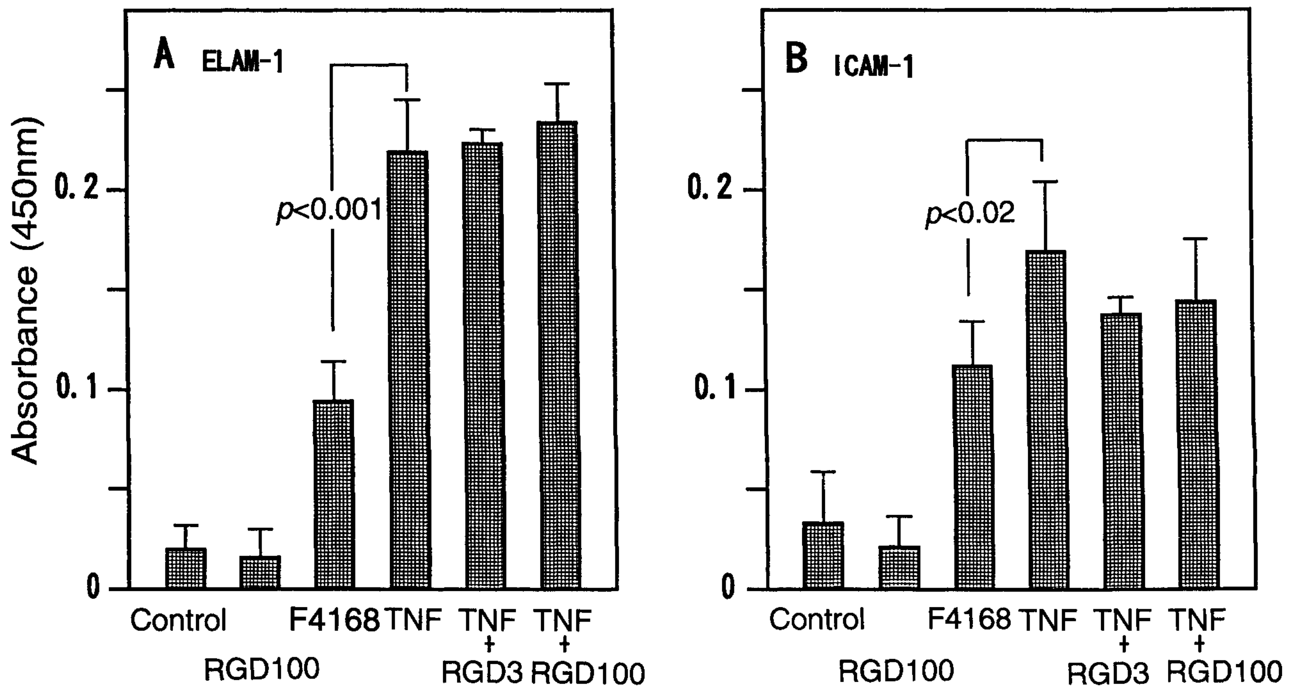

FIG. 5. Effect of GRGDS peptide on TNFα-mediated enhancement of cell adhesion molecules. ELAM-1 (A) or ICAM (B) expression after stimulating of HUVEC with 0.1 ng/ml of TNFα, F4168 or GRGDS peptide (RGD100, 100 pg/ml; RGD3, 3 pg/ml) for 4 h. Bars indicate the mean ± S.D., and significance was determined by Student's *t*-test.

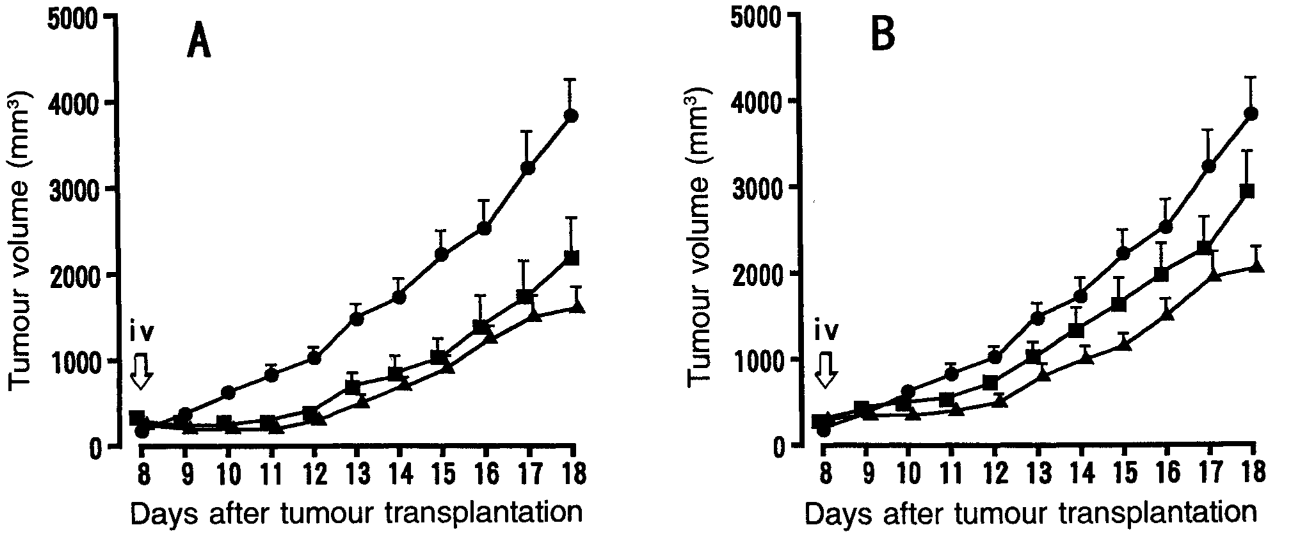

FIG. 6. Growth inhibition against Meth A fibrosarcoma by TNFα or F4168. Meth A cells ($1 \times 10^6$) were transplanted subcutaneously into the back of BALB/c male mice (6 weeks old). After 8 days, when the tumours were 8 mm in mean diameter, TNFα or F4168 was injected intravenously into the tail vein at a dose of 3 μg (A) or 1 μg (B) per mouse. Tumour volumes were calculated by the formula $ab^2/2$, where *a* and *b* represent the long and short diameters of the tumor, respectively. ●, Control; ■, TNFα; ▲, F4168; Bars indicate the mean ± S.E.M. ($n = 5$).

ondary' inflammation. The 'primary inflammation' occurs immediately after TNFα injection and includes haemorrhage, thrombus formation, congestion of capillary vessels and complete loss of blood circulation followed by the necrosis of tumour vasculature.[15] At the same time, adhesion or infiltration of neutrophils or macrophages on vessel walls is promoted, which might result in the destruction of tumour vasculature. These sequential reactions at the early stage are thought to be mainly concerned with the expression of the antitumour activity of TNFα.

On the other hand, following the primary response, a secondary response at the inflammatory site (tumour region) could occur, in which several inflammatory cytokines such as IL-1, IL-6 and TNFα are induced from macrophage or lymphocytes. These inflammatory cytokines induce several cell adhesion molecules which promote the interaction between endothelial cells and leukocytes through their specific receptors.[16–18] Neutrophils accumulated at the inflammatory site release various proteases or reactive oxygen metabolites which cause the destruction of tumour-induced capillary vessels. It is surmised that such 'secondary inflammation' promotes the antitumour activity of TNFα at a lower concentration.

This study indicates that F4168 is less toxic to the intestine than is TNFα. Based on the histopathological findings (Fig. 1 and Table 1), it is speculated that this difference between F4168 and TNFα is accompanied by a varied extent of secondary inflammation, especially the accumulation of activated neutrophils at the inflammatory site. It has been reported that the binding of leukocytes to endothelial cells is mediated by cell adhesion molecules belonging to the selectin or immunoglobulin family.[19–21] The present results show that ELAM-1 or ICAM-1 expression on the endothelial cell surface is up-regulated by TNFα stimulation (Figs 3 and 4), which could be correlated with the enhanced neutrophil binding to endothelial cells (Fig. 2). Similar results have been reported by Pober *et al.*[21–23] In contrast, F4168 induced a much lower level of ELAM-1 or ICAM-1 expression (Fig. 5) and neutrophil binding (Fig. 2) on endothelial cells compared with that of TNFα. Therefore, F4168 should induce neither excessive rolling and infiltration of neutrophils nor excessive secondary inflammation *in vivo*. This may explain why F4168 does not induce serious intestinal injury.

It is of particular note that a synthetic GRGDS pentapeptide has the potential to reduce TNFα mediated ICAM-1 expression (Fig. 5B), because this fact could provide a better understanding of the mechanisms by which F4168 shows much lower inducibility of cell adhesion molecules. Since F4168 (but not TNFα) mediates the adhesion and spreading of endothelial cells onto an inert substrate (data not shown), it is possible that RGD-directed receptor is involved in the binding of F4168 with endothelial cells. As described above, stimulation by F4168 through the TNF receptor up-regulates the expression of adhesion molecules. In contrast, stimulation through RGD receptors (integrins) may reduce the excessive TNF mediated expression of adhesion molecules. Moreover, it is suspected that the RGD domain in F4168 functions more effectively than an RGD oligopeptide, because TNF functions as an effective carrier protein for it. Thus, binding between the RGD and integrin could effectively occur. Another possibility is that truncated F4168, whose TNF activity is abolished as a result of digestion by proteases secreted from activated neutrophils,[24–26] may also express RGD-derived functions more effectively *in vivo*. In addition to the low toxic effect of F4168 on intestinal function, this novel mutein has the same antitumour potential in mice transplanted with Meth A fibrosarcoma (Fig. 6). Therefore, this mutein should be a useful antitumour drug without the side effects of wild-type TNFα.

### References

1. Carswell EA, Old LJ, Kassel RL, Green S, Fiore N, Williamson B. An endotoxin-induced serum factor that causes necrosis of tumors. *Proc Natl Acad Sci USA* 1975; **72**: 3666–3670.
2. Wang AM, Creasey AA, Ladner MB. Molecular cloning of the complementary DNA for human tumor necrosis factor. *Science* 1985; **228**: 149–154.
3. Sugarman BJ, Aggarwal BB, Hass PE, Figari IS, Palladino Jr MA, Shepard HM. Recombinant human tumor necrosis factor-α: effects on proliferation of normal and transformed cells *in vitro*. *Science* 1985; **230**: 943–945.
4. Tracey KJ, Beutler B, Lowry SF. Shock and tissue injury induced by recombinant human cachectin. *Science* 1986; **234**: 470–474.
5. Sun X, Hsueh W, Torre-Amione G. Effects of *in vivo* 'priming' on endotoxin-induced hypotension and tissue injury. *Am J Pathol* 1990; **136**: 949–956.
6. Miyata K, Kato M, Shikama H. A YIGSR-containing novel mutein without the detrimental effect of human TNFα of enhancing experimental pulmonary metastasis. *Clin Exp Metastasis* 1992; **10**: 267–272.
7. Lollini PL, De Giovanni C, Nicoletti G. Enhancement of experimental metastatic ability by tumor necrosis factor-alpha alone or in combination with interferon-gamma. *Clin Exp Metastasis* 1990; **8**: 215–224.
8. Orosz P, Echtenacher B, Falk W, Rüschoff J, Weber D, Männel DN. Enhancement of experimental metastasis by tumor necrosis factor. *J Exp Med* 1993; **177**: 1391–1398.
9. Sun X, Hsueh W. Bowel necrosis induced by tumor necrosis factor in rats is mediated by platelet-activating factor. *J Clin Invest* 1988; **81**: 1328–1331.
10. Kahky MP, Daniel CO, Cruz AB, Gaskill III HV. Portal infusion of tumor necrosis factor increases mortality in rats. *J Surg Res* 1990; **49**: 138–145.
11. Hsueh W, Sun X, Rioja LN, Gonzalez-Crussi F. The role of the complement system in shock and tissue injury induced by tumour necrosis factor and endotoxin. *Immunology* 1990; **70**: 309–314.
12. Kunkel TA, Roberts JD, Zakour RA. Rapid and efficient site-specific mutagenesis without phenotypic selection. In: Wu R, Grossman L, eds. *Methods in Enzymology, Vol. 154*. Orlando, FL: Academic Press, 1987; 367–382.
13. Bradford MM. A rapid and sensitive method for the quantitation of microgram quantities of protein utilizing the principle of protein-dye binding. *Anal Biochem* 1976; **72**: 248–254.
14. Jaffe EA, Nachman RL, Becker CG, Minick CR. Culture of human endothelial cells derived from umbilical veins. *J Clin Invest* 1973; **52**: 2745–2756.
15. Clauss M, Murray JC, Vianna M. A polypeptide factor produced by fibrosarcoma cells that induces endothelial tissue factor and enhances the procoagulant response to tumor necrosis factor/cachectin. *J Biol Chem* 1990; **265**: 7078–7083.
16. Springer TA. Adhesion receptors of the immune system. *Nature* 1990; **346**: 425–434.
17. Lasky LA. Selectins: interpreters of cell-specific carbohydrate information during inflammation. *Science* 1992; **258**: 964–969.
18. Zimmerman GA, Prescott SM, McIntyre TM. Endothelial cell interactions with granulocytes: tethering and signaling molecules. *Immunol Today* 1992; **13**: 93–100.
19. Lawrence MB, Springer TA. Leukocytes roll on a selectin at physiologic flow rates: distinction from and prerequisite for adhesion through integrins. *Cell* 1991; **65**: 859–873.
20. Von Andrian UH, Chambers JD, McEvoy LM, Bargatze RF, Arfors KE, Butcher EC. Two-step model of leukocyte-endothelial cell interaction in inflammation: distinct roles for LECAM-1 and the leukocyte β2 integrins *in vivo*. *Proc Natl Acad Sci USA* 1991; **88**: 7538–7542.
21. Bevilacqua MP, Pober JS, Mendrick DL, Cotran RS, Gimbrone Jr MA. Identification of an inducible endothelial-leukocyte adhesion molecule. *Proc Natl Acad Sci USA* 1987; **84**: 9238–9242.
22. Pober JS, Gimbrone Jr MA, Lapierre LA. Overlapping patterns of activation of human endothelial cells by interleukin 1, tumor necrosis factor, and immune interferon. *J Immunol* 1986; **137**: 1893–1896.
23. Pober JS, Bevilacqua MP, Mendrick DL, Lapierre LA, Fiers W, Gimbrone Jr MA. Two distinct monokines, interleukin 1 and tumor necrosis factor, each independently induce biosynthesis and transient expression of the same antigen on the surface of cultured human vascular endothelial cells. *J Immunol* 1986; **136**: 1680–1687.
24. Van Kessel KPM, Van Strijp JAG, Verhoef J. Inactivation of recombinant human tumor necrosis factor-α by proteolytic enzymes released from stimulated human neutrophils. *J Immunol* 1991; **147**: 3862–3868.
25. Scuderi P, Nez PA, Duerr ML, Wong BJ, Valdez CM. Cathepsin-G and leukocyte elastase inactivate human tumor necrosis factor and lymphotoxin. *Cell Immunol* 1991; **135**: 299–313.
26. Nortier J, Vandenabeele P, Noël E, Bosseloir Y, Goldman M, Lanckman MD. Enzymatic degradation of tumor necrosis factor by activated human neutrophils: role of elastase. *Life Sciences* 1991; **49**: 1879–1886.

ACKNOWLEDGEMENTS. The authors thank Dr T. Tsujimoto (Vice-director of Saiseikai Shiga Hospital) for initial help with HUVEC isolation. The authors also thank Dr Y. Kiho, Director of Biochemistry Research Laboratory and Emeritus Professor Y. Takagi of Kyushu University for helpful discussions, and K. Kawagoe, T. Kawagoe and Y. Mitsuishi for expert technical assistance.

**Received 15 November 1993; accepted 7 December 1993**
